# Targeting JNK by a New Curcumin Analog to Inhibit NF-kB-Mediated Expression of Cell Adhesion Molecules Attenuates Renal Macrophage Infiltration and Injury in Diabetic Mice

**DOI:** 10.1371/journal.pone.0079084

**Published:** 2013-11-18

**Authors:** Yong Pan, Xiuhua Zhang, Yi Wang, Lu Cai, Luqing Ren, Longguang Tang, Jingying Wang, Yunjie Zhao, Yonggang Wang, Quan Liu, Xiaokun Li, Guang Liang

**Affiliations:** 1 Chemical Biology Research Center, School of Pharmaceutical Sciences, Wenzhou Medical University, Wenzhou, Zhejiang, China; 2 Chinese-American Research Institute for Diabetic Complications, Wenzhou Medical University, Wenzhou, Zhejiang, China; 3 Department of Pharmacy, The 1st Affiliated Hospital, Wenzhou Medical University, Wenzhou, Zhejiang, China; 4 Department of Pediatrics, University of Louisville, Louisville, Kentucky, United States of America; 5 Department of Cardiology, The First Hospital of Jilin University, Changchun, Jilin, China; Medical University Innsbruck, Austria

## Abstract

Macrophage infiltration contributes to the pathogenesis of diabetic renal injury. However, the regulatory mechanisms between macrophage infiltration and epithelial cell activation are still unclear. Our previous study found that C66, a novel curcumin analog, was able to inhibit inflammatory cytokine expression in vitro and in vivo. This study further elucidated whether C66 can prevent glucose-induced renal epithelial activation and inflammatory macrophage infiltration by a MAPK/NF-κB medicated mechanism. Our data show that pretreatment with C66 not only significantly reduced high glucose (HG)-induced over-expressions of VCAM-1, ICAM-1 and MCP-1, but also remarkably inhibited NF-κB activation, MAPKs phosphorylation, and subsequently macrophage adhesion in renal epithelial NRK-52E cells. Furthermore, we find that MAPKs, especially JNK, play important roles in HG-induced NF-κB activation, which regulates the over-expression of adhesion molecules in HG-stimulated NRK-52E cells. A molecular docking predicted that C66 may target JNK2, which leads to its anti-inflammatory actions. In vivo, administration of C66 or JNK special inhibitor SP600125 at 5 mg/kg markedly decreased diabetes-induced renal adhesion molecule expression, NF-κB activation, inflammatory cell infiltration, and pathological indexes in the kidneys of diabetic mice. These findings provide a perspective on the renoprotective effects of C66 in diabetes, and outline a novel therapeutic strategy of JNK inhibition for the treatment of diabetic nephropathy.

## Introduction

Findings from both human and animal models of diabetic nephropathy suggest that kidney macrophage accumulation is a major factor of diabetic renal damage [1]. A study of patients with type 2 diabetes indicated that macrophages increased transiently in glomeruli during the progression from mild to moderate glomerulosclerosis [2]. Accumulation of macrophages in diabetic kidneys appears to occur through common recruitment mechanisms, involving increased expression of cell adhesion molecules and chemokines. Studies have identified increased gene expression or protein levels of selectin, intercellular adhesion molecule-1 (ICAM-1), and vascular cell adhesion molecule-1 (VCAM-1), and monocyte chemotactic protein 1 (MCP-1) in the kidneys during the early development of diabetic renal injury both in human beings and animal models [1], [3], [4]. Circulating forms of these molecules have also been detected in the plasma of patients with diabetic nephropathy [5]. Elements in the diabetic milieu have been shown to stimulate expressions of ICAM-1, VCAM-1, and MCP-1 in kidney tissue, which further enhance adhesions of the circulating blood monocytes into glomerulum [6], [7]. Among the intracellular signaling system involved in the regulation of inflammatory and immune responses, mitogen-activated protein kinases (MAPKs) and nuclear factor (NF)-κB pathways are of specific importance [8]. These signaling pathways regulate the gene expressions of pro-inflammatory mediators, including chemokines and adhesion molecules, in a variety of cell types [9]. MAPK pathways constitute extracellular regulated kinase (ERK), c-Jun NH_2_-terminal kinase (JNK), and p38 MAPK. Recent reports showed that p38 and JNK pathways may play important roles in regulating ICAM and MCP-1 expression in high glucose (HG)-induced renal cells and diabetic kidney tissues [10], [11]. In addition, NF-κB has been reported to regulate the gene expressions of adhesion molecules and chemokines in both renal cells and diabetic kidney tissues [12], [13]. Experimental studies have shown that NF-κB blockage by various methods prevents diabetic renal damage [12]–[15]. Despite their significant roles, the crosstalk mechanisms by which MAPKs and NF-κB mediated diabetes-induced macrophage infiltration are unclear.

In our previous studies, we have designed and synthesized a curcumin analogue, (2E,6E)-2,6-bis(2-(trifluoromethyl)benzylidene)cyclohexanone (C66), which exhibited strong inhibitory effect on LPS-induced inflammatory cytokine expression in mouse macrophages [16]. It also exhibited anti-inflammatory actions in HG-stimulated macrophages and renoprotective effects in diabetic rats [17]. This compound is being evaluated in preclinical study as a new renoprotective candidate and the previous results also showed that it has high bioavailability and safety in dogs (unpublished data). In this study, we investigated the preventive effects of C66 on renal epithelial activation and macrophage infiltration in diabetes. Importantly, we gained new insights of MAPK/NF-κB pathways causing diabetic renal macrophage infiltration using C66 and specific inhibitors as small molecule probes.

## Materials and Methods

### Antibodies and Reagents

**Figure 1:**
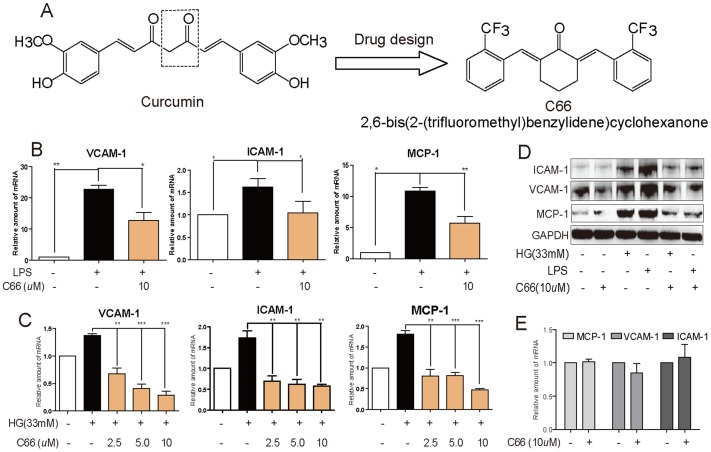
C66 inhibited LPS- and HG-induced mRNA expression of adhesion molecules and chemokines in NRK-52E cells. A) Chemical structures of curcumin and C66. B) and C). NRK-52E cells (2.5×10^6^) were pretreated with C66 at various concentrations (2.5, 5, or10 µM) or DMSO for 2 h, then stimulated with 0.5 µg/mL LPS (B) for 8 h or 33 mM HG (C) for 24 h. Total RNA extraction was performed as described in Materials and Methods. The mRNA levels are expressed as a ratio of β-actin and normalized to controls. D) Cells (2.5×10^6^) were pretreated with C66 at 10 µM or DMSO for 2 h, and then stimulated with 0.5 µg/mL LPS for 12 h or 33 mM HG for 24 h. Total protein extraction was analyzed via western blot. E) Cells were treated with C66 at 10 µM or DMSO for 24 h, and total RNA was extracted for RT-qPCR analysis. Bars represent the mean ± SD of four independent experiments in triplicate (* *p*<0.05, **p<0.01, ***p<0.001).

All antibodies used here were purchased from Santa Cruz (*Santa Cruz technology, CA*). PD98059 (ERK1/2 specific inhibitor), SB203580 (p38MAPK specific inhibitor), SP600125 (JNK1/2 specific inhibitor), and BAY-11-7082 (NF-κB inhibitor) were purchased from Sigma (*St.Louis, MO*). A 3.3-kb cDNA fragment (dominant-negative type) encoding HA-tagged JNKK2 (KM)-JNK1 fusion protein, in which lysine 149 in the ATP domain of the JNKK2 moiety was replaced by methionine, and a vector cDNA (control) were gifts from Prof. Aimin Xu (*Department of Medicine at University of Hong Kong, Hong Kong*). Compound C66 (Figure 1A) was synthesized and characterized as described in our previous publication [16]. C66 was dissolved in DMSO for *in vitro* experiments, and was dissolved in 1% CMCNa for *in vivo* experiments.

### Cell Culture and treatment

NRK-52E cells (*ATCC, CRL-1571, Manassas, VA*) were seeded and grown in DMEM (*Gibco, Eggenstein, Germany*) containing 5.5 mM D-Glucose with 10% FBS, 100 U/mL penicillin, and 100 mg/mL streptomycin, at 37°C in a 5% CO_2_ atmosphere.

### Animal Experiments

Protocols involving the use of animals were approved by the Wenzhou Medical College Animal Policy and Welfare Committee. Male C57BL/6 mice weighing 18–22 g were obtained from Animal Center of Wenzhou Medical College (*Wenzhou, China*). Animals were housed at a constant room temperature with a 12∶12 hour light-dark cycle, and fed with a standard rodent diet and water. The animals were acclimatized to the laboratory for at least 3 days before use. Type 1 diabetes was induced by a single intravenous injection of 150 mg/kg streptozotocin (STZ) in citrate buffer (pH 4.5) at the age of 5 weeks. C57BL/6 mice were randomly divided into three groups with six mice in each group: 1) Non-diabetic control mice (Control group), which received an injection of citrate buffer alone; 2) STZ-induced diabetic mice that received vehicle (1% CMCNa) alone (DM group); 3) STZ-induced diabetic mice that were orally treated with C66 (in 1% CMCNa solution) at a dose of 5 mg/kg every other day starting at day seven after STZ injection for 60 days (DM+C66 5 mg/kg group). Body weight and the levels of blood glucose were checked weekly. Sixty seven days after STZ injection, mice were sacrificed under anesthesia. At the time of sacrifice, blood was collected and serum creatinine levels were determined by an automatic biochemical analyzer (*Hitachi Auto Analyzer 7020, Hitachi Co. Ltd, Japan*). Both kidneys were harvested and weighted. The experimental protocol for the supplemental animal study, in which C66-treated control mice, SP600125-treated control mice, and SP600125-treated DM group have been included, is described in online supporting document.

### Primary rat peritoneal macrophage (RPM) preparation and culture

Sprague-Dawley (SD) rats aged 6 weeks were obtained from Animal Center of Wenzhou Medical College. The RPMs were isolated and cultured using the previously described method [17].

### Renal histology and Immunohistochemistry

Kidneys were fixed in 4% paraformaldehyde and embedded in paraffin. The paraffin sections (5 µm) were dehydrated and stained using Masson Trichrome stain. After deparaffinization and rehydration, 5 µm renal sections were treated with 5% H_2_O_2_ for 10 min and with 3% BSA for 30 min. Slides were incubated overnight at 4°C with anti-CD68 antibody , then incubated with fluorescein isothiocyanate (FITC)-labelled goat anti-rabbit IgG for 1 h at room temperature. Following staining the nucleus with DAPI for 5 min, the images were viewed by a fluorescence microscope (400×, *Nikon, Tokyo, Japan*).

### Real-time quantitative PCR

NRK-52E cells or kidney tissues (50–100 mg) were homogenized in TRIZOL (*Invitrogen, Carlsbad, CA*) for extraction of RNA. Both reverse transcription and quantitative PCR were carried out using a two-step M-MLV Platinum SYBR Green qPCR SuperMix-UDG kit (*Invitrogen, Carlsbad, CA*). Eppendorf Mastercycler eprealplex detection system (*Eppendorf, Hamburg, Germany*) was used for q-PCR analysis. The primers of genes described in Table S1 in File S1 were synthesized from Invitrogen (*Invitrogen, Shanghai, China*), and the amount of each gene was determined and normalized to the amount of β-actin.

### Preparation of nuclear extracts

Nuclear protein extractions from NRK-52E cells or kidney tissues (50–100 mg/kidney) were prepared according to the method described in our previous report [18].

### Western blotting

Cells or homogenated kidney tissues were lysated. The protein concentrations in all samples were determined by using the Bradford protein assay kit (*Bio-Rad, Hercules, CA*). Lysates were separated by SDS-PAGE electrophoresis, and electrotransferred to a 0.22 µm polyvinyldene difluoride membrane. After blocked in TBS containing 5% non-fat milk for 1.5 h at room temperature, the membranes were incubated with different primary antibodies overnight at 4°C. Following TBST wash, immunoreactive bands were detected by incubating with secondary antibody conjugated with horseradish peroxidase for 1 h. Immunoreactive bands were visualized by using ECL kit (*Bio-Rad, Hercules, CA*).

### Transient transfection

NRK-52E cells were incubated for 6 h in 1 mL of serum-free medium containing 10 µL of Lipofectamine 2000 reagent (*Invitrogen, CA*) and 2.5 µg of dominant negative JNK (dn-JNK) or vector. After 24-h or 48-h incubation in complete medium, the cells were treated with HG.

### Molecular modeling

Docking simulation of indicated compound with protein was carried out with the program Tripos molecular modeling packages Sybyl-x.v1.1.083 (*Tripos, St. Louis, MO*). The crystal structure of p38a, p38b, ERK2, JNK1, and JNK2 was obtained from Protein Data Bank (p38a:1A9U; p38b:3GP0; ERK2:1ERK; JNK1:1UKI; JNK2:3NPC). The ligand-binding groove on proteins was kept rigid, whereas all torsible bonds of ligands were set free to allow flexible docking to produce more than 100 structures. Final docked conformations were clustered within the tolerance of 1 Å root-mean-square deviation.

### Statistical analysis

Data were collected from repeated experiments and were presented as mean ± SD. Student's *t* test and ANOVA in GraphPad Pro 5.0 (*GraphPad, San Diego, CA*) were used for analysing the differences between sets of data. Differences were considered to be significant at *P*<0.05. All experiments were repeated at least three times.

## Results

### C66 reduced LPS- and glucose-induced adhesion molecule mRNA expressions in NRK-52E cells

Proximal tubular epithelial cells play a crucial role in producing chemokines and cell adhesion molecules for the recruitment, retention, and activation of inflammatory infiltrating cells into kidneys. We used NRK-52E cells, a well-characterized line of rat proximal tubular epithelial cells, to investigate the beneficial effects of C66. As shown in Figure 1B, 0.5 µg/mL LPS significantly increased production of VCAM-1, ICAM-1 and MCP-1. Pretreatment with C66 at 10 µM significantly reduced such changes (*P*<0.05). Similar results were observed in the HG-stimulated cells. Figure 1C shows that HG (33 mM) significantly increased the expression of VCAM-1, ICAM-1 and MCP-1 in NRK-52E cells, while treatment of C66, significantly inhibited these productions (*P*<0.01). Also, both LPS and HG induced the expression of these three proteins, which were remarkably inhibited by the pretreatment with C66 at 10 µM (Figure 1D). Consistent with previous reports [6], [7], LPS exhibited stronger regulation in these gene expressions than HG stimulation in renal cells (Figure 1B–1D). Our previous results show that osmotic changes treatment with mannitol did not change these inflammatory profiles using mannitol as a control [17]. In addition, C66 itself did not cause any change of gene expressions of VCAM-1, ICAM-1, and MCP-1 (Figure 1E). In summary, C66 was able to reduce expressions of adhesion molecules and chemokines *in vitro*.

### Suppression of HG-induced gene expressions by C66 is associated with MAPK/NF-κB inactivation

**Figure 2:**
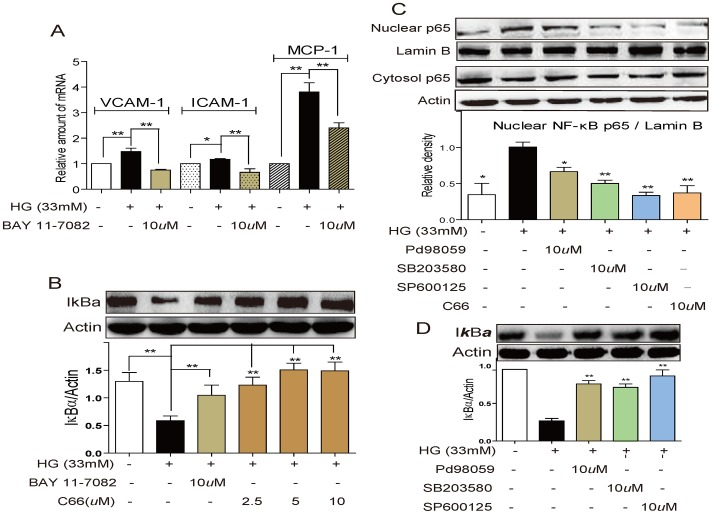
The inhibitory activity of C66 against expression of adhesion molecules is mediated by MAPK and NF-κB inactivation. A). NF-κB inhibitor reduced the mRNA expression of adhesion molecules and chemokines. B). C66 reversed HG-induced IκBα degradation in NRK-52E cells. C) Western blot analysis of nuclear and cytosolic protein (normalized optical density as percentage of low glucose-treated group) showed both C66 and specific inhibitors of MAPKs inhibited HG-induced NF-κB p65 nuclear translocation. D) MAPK inhibitors down-regulated the HG-induced NF-κB activation. RNA levels are expressed as a ratio of β-actin, normalized to controls. Actin was used as a loading control. Bars represent the mean ± SD of more than three independent experiments (*p<0.05, **p<0.01, ***p<0.001).

NF-κB is of importance in the transcriptional regulation of different genes, including VCAM-1, ICAM-1, and MCP-1 [9]. We pretreated the HG-incubated cells with a specific NF-κB inhibitor BAY 11-7082. As shown in Figure 2A, BAY 11-7082 significantly reduced HG-induced VCAM-1, ICAM-1, and MCP-1 expressions (*P*<0.01). IκB is the inhibitory subunit of NF-κB in the cytosol. The IκBα degradation leads to NF-κB p65 translocation from the cytosol to the nucleus. Hence we measured the effects of C66 on IκBα degradation and p65 translocation in NRK-52E cells. Figure 2B showed that pretreatment with C66 dose-dependently reversed the HG-induced degradation of IκBα (*P*<0.01). As shown in Figure 2C, HG accelerated NF-κB p65 nuclear translocation, whereas the C66-pretreatment decreased this translocation (*P*<0.01). These data indicated that NF-κB signaling plays a role in HG-induced adhesion molecule and chemokine over-expression, and that the anti-inflammatory effect of C66 is associated with its NF-κB inactivation.

Increased phosphorylation of MAPKs is present in both intrinsic renal cells and infiltrating leukocytes in glomerulonephritis, and correlates with severity of renal dysfunction and structural changes [19]. Here, we examined the possible involvement of MAPKs in NF-κB activation in NRK-52E cells. We pretreated the HG-incubated cells with Pd98059 (ERK inhibitor), SB203580 (p38 inhibitor) and SP600125 (JNK inhibitor), respectively, and analyzed the nuclear p65 protein and cytosol IκBα levels by western blot. The results in Figure 2C indicated that all these inhibitors significantly reduced HG-induced increase of nuclear p65 protein levels in NRK-52E cells (p<0.05). Similar result was also observed in the reversal of IκBα degradation by three inhibitors (p<0.01, Figure 2D), indicating that ERK, p38 and JNK signaling pathways mediate the HG-induced NF-κB activation. Interestingly, among these three pathways, JNK shows strongest effects on NF-κB activation (Figures 2C and 2D).

### MAPKs, especially JNK, may mediate C66's attenuation on HG-induced inflammation

**Figure 3:**
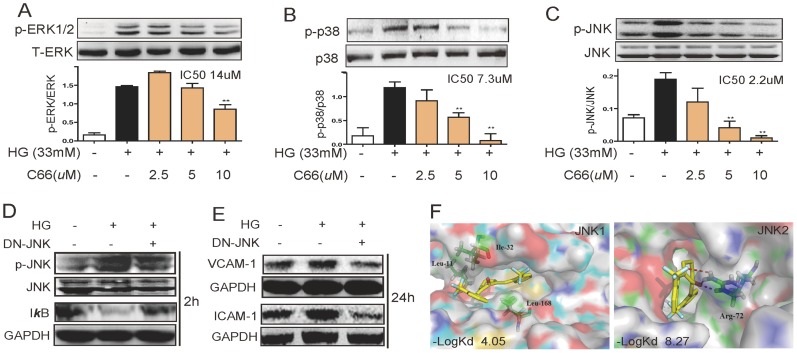
JNK may mediate the beneficial effects of C66. **A-C**). C66 inhibited the phosphorylation of ERK1/2 (A), p38 (B) and JNK (C). The bar graphs show the normalized optical density as percentage of low glucose-treated group (*p<0.05, **p<0.01). D&E). NRK-52E cells were transfected with dn-JNK plasmid or vector. Such process corrected glucose induced IκB activation (D) and reduced VCAM-1 and ICAM-1 productions (E). F). Two representative figures show molecular docking between C66 and JNK1 or JNK2, respectively. The binding scores (-log Kd) between C66 and JNKs were obtained using the docking software.

We then tested the effects of C66 on MAPK activation in HG-stimulated NRK-52E cells. As shown in Figures 3A–3C, HG incubation increased the phosphorylation of MAPKs by 1.8–7.7 folds, while C66 pretreatment significantly inhibited HG-induced ERK1/2, p38 and JNK phosphorylation in dose-dependent ways. Among these three pathways, three MAPK pathways exhibited different sensitivities towards C66 treatment, and JNK activation seems to be inhibited by C66 to the largest extent with an IC_50_ of 2.2 µM.

Thus, we supposed that JNK may be the most critical mediator in HG-induced NF-κB activation and C66's anti-inflammatory actions. To exclude possible non-specific inhibition by the chemical inhibitor, NRK-52E cells were also infected with a dn-JNK and then stimulated with HG for 2 h. Figure 3D showed that dn-JNK significantly prevented the HG-induced degradation of IκB. Importantly, incubation of HG for 24 h increased the expressions of ICAM-1 and VCAM-1, which was also remarkably prevented by JNK inactivation (dn-JNK) (Figure 3E). Hence JNK activation is directly involved in mediating HG-induced NF-κB activation and inflammation.

Above evidences, especially an IC_50_ of 2.9 µM, implied that JNK may be a molecular target of C66. Thus, we proposed a docking model of C66 and special inhibitors with the crystal structures of JNK1 and JNK2, respectively (Figure 3F). The Surflex-Dock scores of ligand-protein dockings were expressed in −logK_d_ unit. C66 exhibited less binding affinity to p38a, p38b, ERK1, ERK2, and JNK1 than their special inhibitors (data not shown), respectively, while the docking between C66 and JNK2 displays a much higher affinity (−LogK_d_: 8.27) than that between SP600125 and JNK2 (−LogK_d_: 5.30), indicating that C66 exhibit a much higher JNK2-binding affinity than SP600125. Interestingly, the binding scores of C66 with these kinases are positively correlated with the IC_50_ values of C66 against the kinase phosphorylation (Figure 3A–C). A higher binding score leads to a lower IC_50_ value, indicating a relatively correct docking model. As shown in Figure 3F, C66 was buried inside JNK2 pocket and achieved two hydrogen bonds with Arg72 at the opening rims of the pocket. C66 also interacted with the hydrophobic residues in this pocket in the most energetically favorable simulation. Based on the *in vitro* and *in silico* data, it may be inferred that C66 directly targets JNK2 with a low selectivity on p38 kinase and a high selectivity on ERK.

### C66 inhibited epithelial-macrophage adhesion in vitro

**Figure 4:**
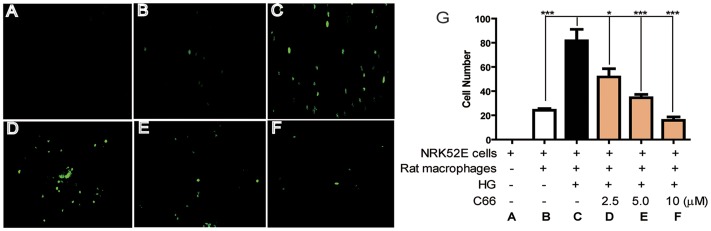
C66 dose-dependently inhibited glucose-induced epithelial-macrophage adhesion (green spots showed the location of macrophages in NRK-52E cell cultured plate. The bar graph represents the number of attached macrophages in five randomly selected optical fields per well, and expressed as mean ± SD of three independent experiments (*p<0.05, ***p<0.001).

We next performed macrophage adhesion assays to examine its inhibitory effect on epithelial-macrophage adhesion *in vitro*. As shown in Figure 4, HG stimulation significantly increased macrophage adhesion to NRK-52E cells. Such HG-induced adhesion were diminished by C66 treatment, in a dose-dependent manner (Figure 4G).

### Administration of C66 decreased renal expressions of VCAM-1, ICAM-1, and MCP-1, and inhibited NF-κB activation in diabetic kidneys

**Figure 5:**
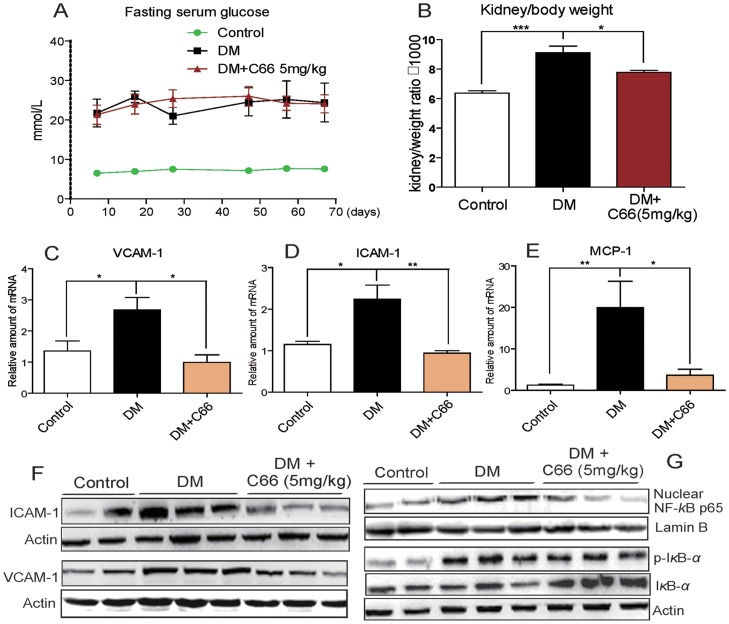
C66 treatment decreased renal expression of adhesion molecules, chemokines and NF-κB activation in diabetic mice. A) Diabetes induced increased blood glucose levels were not affected by C66 treatment. B) Kidney/body weight ratios in diabetic mice were improved by C66 treatment. C–E). Increased renal mRNA expressions of VCAM-1 (C), ICAM-1 (D), and MCP-1 (E) in diabetic mice were prevented by C66 treatment. RNA levels (mean ± SD) are expressed as a ratio of β-actin n = 6/group (* *p*<0.05, **p<0.01). F) Western blot analysis of extracted proteins from the nuclear and cytoplasmic fractions showing diabetes-induced increased expressions of VCAM-1 and ICAM-1, and NF-κB activation were prevented by C66. (DM = diabetic mice).

To validate the beneficial functions of C66 *in vivo*, a model of STZ-induced diabetic mice was used. Seven days after STZ injection, all mice developed overt diabetes (blood glucose >20 mM). The mice in DM+C66 group were orally treated with C66 at 5 mg/kg/day for 2-month. Figure 5A showed that there was no significant difference in blood glucose levels between the DM-control and DM+C66-treated group, indicating that C66 treatment did not affect blood glucose level in the diabetic mice. Sixty days after STZ induction, mice were sacrificed and the kidneys were analyzed. As shown in Figure 5B, kidney weight in relation to body weight was significantly increased in the DM group (*p*<0.01), whereas a decrease was observed in C66 treated groups compared with DM group (*p*<0.05). Consistent to the *in vitro* data, significantly increased expressions of VCAM-1, ICAM-1, and MCP-1 were observed in DM group (*p*<0.05), while treatment with C66 markedly reduced the levels of these molecules (*p*<0.05, Figure 5C–5E). Western blot analysis also revealed similar results in the protein level (Figure 5F). In a supplementary animal experiment, oral administration with C66 or SP600125 at 5 mg/kg for 12 weeks also significantly inhibited hyperglycemia-induced renal over-expression of VCAM-1, ICAM-1, and MCP-1 at the levels of both mRNA and protein (Figure S1 in File S1). The beneficial effects of SP600125 also validated the important role of JNK in the development of diabetic renal inflammation. Furthermore, administration with C66 for 2 months significantly inhibited diabetes-induced renal p65 nuclear translocation (*p*<0.01) and renal IκBα phosphorylation and degradation (*p*<0.01) when compared to those in DM group (Figure 5G). These data confirm that C66 administration decreased renal expression of adhesion molecules and chemokines coupled with its inhibitory effects on NF-κB activation.

### Administration of C66 suppressed inflammatory cell infiltration and attenuated renal injury in diabetic mice

**Figure 6:**
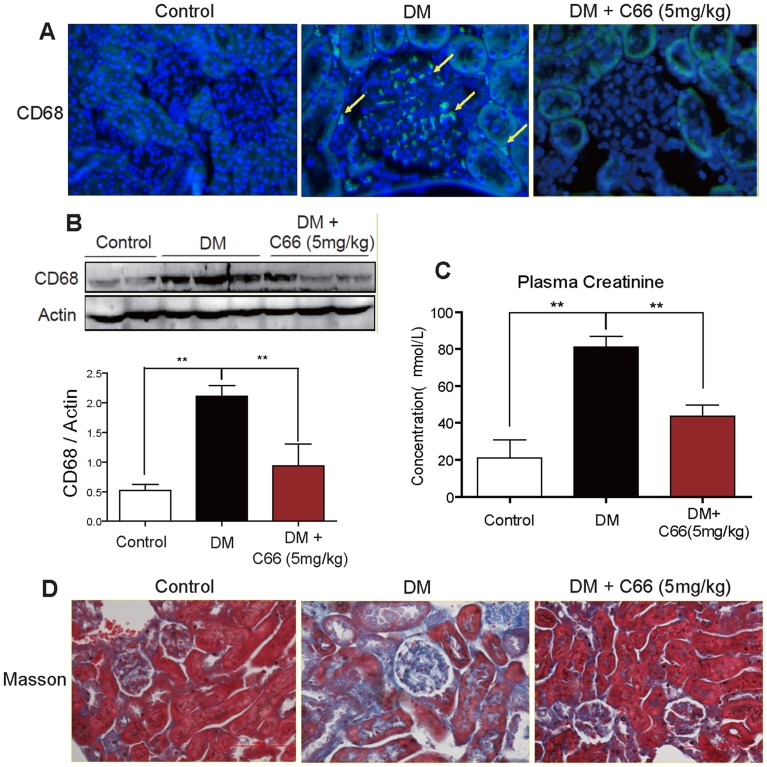
Systemic administration of C66 reduced interstitial inflammatory cell infiltration and reduced renal injury in the kidneys in the diabetic mice. A) Representative photomicrographs showing Macrophage infiltration by immunochemical analysis using anti-CD68 antibodies in the renal tissues (arrows indicate stained macrophages). B) Western blot using anti-CD68 antibodies and densitometric analyses of the renal tissues confirmed such notion. n = 6/group, CD68 levels were normalized to actin (*p<0.05, **p<0.01). D) Increased plasma creatinine levels in diabetes were normalized by C66 administration (**p<0.01). F) Representative masson trichrome stained kidneys (original magnification ×400 for all) showing diabetes induced increased glomerular fibrosis were corrected by C66 treatment (DM = diabetic mice).

Immunohistochemistry staining for CD68 was performed using kidney tissues from C66-treated or non-treated diabetic mice to observe focal interstitial and glomerular macrophage infiltration. As shown in Figure 6A, the diabetic mice (DM group) had a significant increase of CD68 positive macrophages both in interstitial and glomerular areas while renal macrophages were hardly found in the control mice and C66-treated diabetic mice. The beneficial results were further confirmed by western blot using CD68 antibody (*p*<0.01, Figure 6B), showing that C66 significantly reduced renal macrophage infiltration in diabetic mice.

The anti-inflammatory activity of C66 in diabetic kidney may contribute to its protection of diabetic renal injury. Serum creatinine is one of the hallmarks of renal injury. Figure 6C and Figure S2A in File S1 showed that after STZ induction, the mean serum creatinine level of the DM group was significantly higher than those of the control (*p*<0.01) and the C66- or SP600125-treated diabetic mice (*p*<0.05). H&E staining confirmed the renoprotection of C66 and SP600125 from renal structural disorders in diabetic mice (Figure S2B in File S1). Furthermore, the masson trichrome staining further validated the renal histological improvement in C66-treated diabetic mice. As shown in Figure 6D and Figure S2C in File S1, increased fibrosis (stained blue area) was detected in the diabetic kidney (DM group); while fibrosis was particularly severe in the glomerulus, where the majority of infiltrating immunocytes were found. However, C66- and SP600125-treated group demonstrated significantly decreased fibrosis.

## Discussion

Elements of the diabetic milieu can promote macrophage recruitment by inducing renal expression of MCP-1 and cell adhesion molecules. Furthermore, infiltrated macrophages in the inflamed kidney can produce profibrotic cytokines such as TGF-β, which plays a crucial role in progressive renal fibrosis [20]. Thus, therapeutic intervention with mycophenolate mofetil, mizoribine and rapamycin that reduced macrophage accumulation and recruitment in rodent diabetic kidneys were demonstrated to be beneficial for the treatment of diabetic nephropathy [21]–[24].

It is reported that ICAM-1, VCAM-1, and MCP-1 play important roles in the pathogenesis of diabetic nephropathy through inducing inflammatory cell infiltration [25], [26]. Compounds, such as colchicines [20], beraprost sodium [27], and mycophenolate mofetil [28], have been demonstrated to ameliorate inflammatory cell infiltration in diabetic nephropathy by inhibiting MCP-1 expression. In this study, we observed that VCAM-1, ICAM-1, and MCP-1 expression were increased in HG-incubated NRK-52E cells and in experimental diabetic renal injury, which was associated with inflammatory cell infiltration and adhesion, and renal fibrosis. Both *in vitro* and *in vivo*, these increases under high glucose conditions were attenuated by the treatment with a novel anti-inflammatory compound C66. Taken together, the anti-inflammatory effect of C66 in diabetic renal injury may partly be attributed to the suppression of VCAM-1, ICAM-1, and MCP-1 expression in renal epithelial cells.

We further investigated the transcriptional mechanism by which C66 inhibited HG-induced adhesion molecule expression. NF-κB is the most important transcription factor involved in the pathophysiology of diabetic nephropathy. NF-κB activation has been demonstrated in renal tissue of animals with streptozotocin-induced diabetes [12]. As expected, our experiments also observed the renal IκB degradation and p65 nuclear translocation in response to HG stimulation in cultured renal epithelial cells and in diabetic mice. NF-κB is thought to cause renal inflammatory process by regulating gene expression of cytokines, chemokines, and adhesion molecules in chronic renal diseases [9]. Previous studies showed that the expressions of VCAM-1 and ICAM-1 are partly NF-κB dependent [9]. Two independent groups also reported that NF-κB blockade resulted in an inhibition of MCP-1 gene expression in epithelial cells and subsequent macrophage infiltration [29], [30]. Our results showed that blockage of NF-κB by the specific inhibitor BAY 11-7082 or C66 significantly attenuated the HG-induced expression of ICAM-1, VCAM-1, and MCP-1 in renal epithelial cells. The reduction of these molecule expressions by C66 administration is also accompanied with the inactivation of NF-κB in the kidney in diabetes. Hence, it seems reasonable to assume that C66 may exert its anti-inflammatory action by inhibiting NF-κB signaling pathway.

All of three subfamilies of MAPKs, especially JNK and p38, have been reported to be activated in response to inflammatory and stressful stimuli, including HG. Previous studies demonstrated that the exposure to glucose activated both ERK and p38, but not JNK in rat aortic smooth muscle cells [31], rat mesangial cells [32], and bovine aortic endothelial cells [33]. Another study showed that HG was capable of inducing JNK but not ERK and p38 in HUVECs [34]. This study showed, for the first time, that ERK, p38, and JNK were all activated by HG in NRK-52E cells. This result is consistent with recent reports showing activation of p38 and JNK in human diabetic nephropathy [35], [36].

One of the interesting findings in this study was that all three subfamilies of MAPKs were involved in HG-induced NF-κB activation to different extent (Figure 2). Although there are few reports regarding the crosstalk between MAPKs and NF-κB, they seem to modulate the inflammatory cytokine expression independently in HG-induced renal cells and diabetic kidneys. Some literatures have shown that p38 and JNK activation may up-regulate NF-κB by phosphorylation of IKK-β in LPS-stimulated macrophages [37], [38]. Our data showed that blockade of MAPKs by the specific inhibitors attenuated the HG-induced activation of NF-κB, at the levels of both IκBα and p65.

On the other hand, it is worthy to be noted that JNK inhibitor was found to be the strongest interferent against HG-induced NF-κB activation. These data implied a special importance of JNK in HG-induced renal inflammation. We presumed that JNK is an important upstream regulator of NF-κB activation and subsequent cytokine expression in NRK-52E cells. DN-JNK was used to confirm the important role of JNK, which significantly attenuated the HG-induced NF-κB activation and downstream effects (Figure 3D). These data further validated that JNK is a critical upstream molecule of NF-κB and plays an important role in HG-induced renal inflammation. To our knowledge, this is the first report to find JNK-mediated VCAM-1 and ICAM-1 expression induced by HG, indicating the importance of JNK in HG-induced renal cytokine production and macrophage infiltration (Figure 3E).

In this study C66 showed dose-dependent inhibition on HG-induced MAPK phosphorylation (Figure 3), and especially, it exhibited strongest inhibition on JNK phosphorylation with the lowest IC_50_ of 2.2 µM. Computer-assisted molecular docking further validated our hypothesis that C66 may target MAP kinases. C66 was predicted to possess a 288-fold stronger binding affinity to JNK2 than SP600125 at the molecular level. The binding abilities (−Log Kd) of C66 with these three MAPK family kinases are also consistent with its actual inhibitory activities. Although the actual binding needs to be investigated using a series of biochemical experiments, these data indicated a molecular target (JNK2) for the biological effects of C66, and more importantly, supported the critical role of JNK in regulating HG-induced inflammation (Figure 3F). Furthermore, after administrated with C66 or SP600125 for 12-week, diabetic mice exhibited similar attenuation in renal inflammation and pathology (Figures S1 and S2 in File S1). These data confirmed the same pharmacological effects of C66 and JNK specific inhibitor SP600125, indicating that C66 may exert the renoprotective actions via targeting JNK.

C66, developed by our lab in the past five years, is a promising agent for the treatment of diabetic nephropathy. It is being in the later stage of preclinical study and shows good perspective. The *in vivo* mechanistic insights here demonstrated the pharmacological pathway in the anti-inflammatory actions of C66. *In vivo*, C66 treatment did not decrease the STZ-caused hyperglycemia, indicating the beneficial actions of C66 could be attributed to its anti-inflammatory effects (Figures 5, and Figure S1 in File S1). Consistent to the *in vitro* data, it was found that treatment of C66 in diabetic mice could inhibit the NF-κB activation, and then reduce the productions of ICAM-1, VCAM-1, and MCP-1 in renal tissues. Finally, we found that C66 treatment significantly reduced infiltrating renal macrophages, and attenuated circulating creatinine and pathological indexes of renal injury in the diabetic mice (Figure 6, and Figure S2 in File S1).

## Conclusion

In summary, this study demonstrated that JNK is one of the important mediators in HG-induced NF-κB activation and macrophage infiltration in diabetic kidney. A schematic illustration for the prevention of C66 from HG-induced macrophage infiltration and renal injury was shown in Figure S3 in File S1. A synthetic curcumin analogue (C66), targeting JNK2, could decrease HG-induced inflammatory injury both *in vitro* and *in vivo* via the mechanism involving NF-κB and macrophage infiltration, indicating that C66 is a potential candidate for the treatment of diabetic nephropathy.

## Supporting Information

File S1 **A supplemental in vivo study to evaluate the renal-protective effects of C66 in STZ-induced diabetic mice.** File S1 consists of Materials and Methods, and Supplemental Table and Figures (Table S1, Figure S1–S3). **Table S1** **in File S1. Primer sequences for real-time quantitative PCR.** **Figure S1 in File S1.** **C66 and SP600125 treatment attenuated the over-expression of adhesion molecules in diabetic mouse kidneys.** **A–C.** The total RNA in kidney tissues of six groups in the supplemental in vivo study was extracted respectively, and was performed for RT-qPCR analysis to detect the mRNA level of VCAM-1 (A), ICAM-1 (B), and MCP-1 (C). The mRNA levels (mean ± SD) are expressed as a ratio of β-actin. n = 5–8/group). D–F. The total protein in kidney tissues of six groups in the supplemental in vivo study was extracted respectively, and was performed for Western blotting analysis to detect the protein level of VCAM-1 (D), ICAM-1 (E), and MCP-1 (F). The column figures show the normalized optical density from the blot data (4–6 mice in each group, * p<0.05, **p<0.01, v.s. DM group). **Figure S2 in File S1.** **C66 and SP600126 treatment decreased renal injuries in diabetic mice in the supplemental in vivo study.** A. Plasma creatinine levels were determined and normalized by that of one mouse in Con group (*p<0.05). B. H&E staining was used for analysis of histological abnormalities. Six representative figures of histological abnormalities are shown. C. Masson staining was used for analysis of glomerular fibrosis. Six representative figures are shown (×400). **Figure S3 in File S1.**. **A schematic illustration for the prevention of C66 from HG-induced macrophage infiltration and renal injury.**
(DOC)
