# Supplementary material for: Targeting JNK by a New Curcumin Analog to Inhibit NF-kB-Mediated Expression of Cell Adhesion Molecules Attenuates Renal Macrophage Infiltration and Injury in Diabetic Mice
Source: PLoS One. 2013 Nov 18;8(11):e79084. doi: 10.1371/journal.pone.0079084 (PMC3832497; doi:10.1371/journal.pone.0079084)
Supplement: File S1 — A supplemental in vivo study to evaluate the renal-protective effects of C66 in STZ-induced diabetic mice. File S1 consists of Materials and Methods, and Supplemental Table and Figures (Table S1, Figure S1–S3). Table S1 in File S1. Primer sequences for real-time quantitative PCR. Figure S1 in File S1. C66 and SP600125 treatment attenuated the over-expression of adhesion molecules in diabetic mouse kidneys. A–C. The total RNA in kidney tissues of six groups in the supplemental in vivo study was extracted respectively, and was performed for RT-qPCR analysis to detect the mRNA level of VCAM-1 (A), ICAM-1 (B), and MCP-1 (C). The mRNA levels (mean ± SD) are expressed as a ratio of β-actin. n = 5–8/group). D–F. The total protein in kidney tissues of six groups in the supplemental in vivo study was extracted respectively, and was performed for Western blotting analysis to detect the protein level of VCAM-1 (D), ICAM-1 (E), and MCP-1 (F). The column figures show the normalized optical density from the blot data (4–6 mice in each group, * p<0.05, **p<0.01, v.s. DM group). Figure S2 in File S1. C66 and SP600126 treatment decreased renal injuries in diabetic mice in the supplemental in vivo study. A. Plasma creatinine levels were determined and normalized by that of one mouse in Con group (*p<0.05). B. H&E staining was used for analysis of histological abnormalities. Six representative figures of histological abnormalities are shown. C. Masson staining was used for analysis of glomerular fibrosis. Six representative figures are shown (×400). Figure S3 in File S1.. A schematic illustration for the prevention of C66 from HG-induced macrophage infiltration and renal injury. (DOC) [file pone.0079084.s001.doc]

**Supporting Information**

**Targeting JNK by a new curcumin analog to inhibit NF-kB-mediated expression of cell adhesion molecules attenuates renal macrophage infiltration and injury in diabetic mice**

Yong Pan^a,b,*^, Xiuhua Zhang^c,*^, Yi Wang^a,*^, Lu Cai^b,d^, Luqing Ren^a^, Longguang Tang^a^, Jingying Wang^a^, Yunjie Zhao^a^, Yonggang Wang^d,e^, Quan Liu^d,e^, Xiaokun Li^a,b^, and Guang Liang^a, #^

*^a^ Chemical Biology Research Center, School of Pharmaceutical Sciences, Wenzhou Medical University, Wenzhou, Zhejiang, 325035, P. R. China,*

*^b^ Chinese-American Research Institute for Diabetic Complications, Wenzhou Medical University, Wenzhou, Zhejiang, 325035, P. R. China,*

*^c^ Department of Pharmacy, The 1^st^ Affiliated Hospital, Wenzhou Medical University, Wenzhou, Zhejiang,325035, China*

*^d^ Department of Pediatrics, University of Louisville, Louisville, Kentucky, 40202, USA,*

*e00000000000000000000000000000000000000000000000000000000000000000000000000000000000000000000000000000000000000000000000000000000e*

*ingying Wang, 1102452 via targeting JNK2.lidated the important role of JNK in diabetic nephropathy. 000000000000000000000000000The First Hospital of Jilin University, 71 Xinmin Street, Changchun,* *130021, China*

**A supplemental *in vivo* study to evaluate the renal-protective effects of C66 in STZ-induced diabetic mice**

**Materials and Methods**

**Reagents**

All antibodies used here were purchased from Santa Cruz (*Santa Cruz technology, CA*). SP600125 (JNK1/2 specific inhibitor) was purchased from Sigma (*St.Louis, MO*). C66 was dissolved in 1% CMCNa for *in vivo* experiment.

**Animals**

Protocols used for all animal studies were approved by the Wenzhou Medical College Animal Policy and Welfare Committee (Approved documents: 2009/APWC/0031). Male C57BL/6 mice, weighing 18-22 g with 8 weeks of age, were obtained from Animal Center of Wenzhou Medical College (*Wenzhou, China*). Animals were housed in 22 °C with a 12:12 h light/dark cycle; water and mouse standard diet were consumed. To induce diabetes, mice were treated with a single intraperitoneal injection of streptozotocin (STZ; 150 mg/kg in citrate buffer, pH=4.5), while the control animals received the same volume of citrate buffer. The blood glucose level was monitored on day 3 and 7 from the STZ injection with a glucometer. Seven days after STZ injection, mice with fasting-blood glucose >12 mmol/L were considered diabetic. All mice had free access to food and water at all times. Diabetic mice randomly divided into three groups (n=8): diabetic mice (DM+vehicle), C66-treated diabetic mice (DM+C66) and SP600125-treated diabetic mice (DM+SP). Besides, another three control groups were set as C66- and SP-treated control mice (Con+vehicle, Con+C66, and Con+SP). Then C66 or SP at 5 mg/kg were orally given once every 2 days for 12-week. The DM group and age-matched control group were received 1% CMCNa solution alone in the same schedule as the treated DM groups. Animals were sacrificed under sodium pentobarbital anaesthesia. After mice killed, kidney tissues were embedded in 4% paraformaldehyde for pathological analysis and/or snap-frozen in liquid nitrogen for gene and protein expression analysis. In addition, the blood was collected from the right ventricle using a heparin-containing syringe with a needle at the time of death.

**Real-time quantitative PCR**

Total RNA was isolated from kidney tissues (50-100 mg) using TRIZOL (*Invitrogen, Carlsbad, CA*). Reverse transcription and quantitative PCR (RT-qPCR) were performed using M-MLV Platinum RT-qPCR Kit (*Invitrogen, Carlsbad, CA*). Real-time quantitative PCR was carried out using the Eppendorf Realplex_4_ instrument (*Eppendorf, Hamburg, Germany*). Primers of genes including VCAM-1, ICAM-1, MCP-1, and β-actin were synthesized from Invitrogen (*Invitrogen, Shanghai, China*). The primer sequences used were shown in Table S1. The relative amount of each gene was normalized to the amount of β-actin.

**Western blotting**

Kidney tissues lysate homogenates were prepared. Protein samples (30 – 80 μg) were subjected to 10% sodium dodecyl sulfate-polyacrylamide gel electrophoresis, and transfered onto polyvinyldene ﬂuoride membrane (*Bio-Rad Laboratory, Hercules, CA*). After blocked in blocking buffer (5% milk in tris-buffered saline containing 0.05% Tween 20) for 1.5 h at room temperature, membranes were incubated with different primary antibodies overnight at 4 °C. Then membranes were washed in TBS-T and reacted with secondary horseradish peroxidase-conjugated antibody (*Santa Cruz, CA*; 1:5000) for 1-2 h at room temperature. Antigen-antibody complexes were then visualized using enhanced chemiluminescence reagents (*Bio-Rad, Hercules, CA*). The density of the immunoreactive bands was analyzed using Image J software (*NIH, Bethesda, MD*).

**Histopathology**

Kidneys were fixed in 4% paraformaldehyde solution, embedded in paraffin, and sectioned at 5 µm. After dehydration, sections were stained with Hematoxylin and Eosin (H&E). To evaluate the histopathological damage, each image of sections was obtained using a light microscope (400× amplification; *Nikon Tokyo. Japan*).

**Masson staining**

Kidneys were fixed in 4% paraformaldehyde and embedded in paraffin. The paraffin sections (5 µm) were dehydrated and stained with Masson Trichrome to evaluate the collagen collection. The stained sections then were viewed by a Nikon fluorescence microscope (400×amplification; *Nikon Tokyo, Japan*).

**Supplemental Table and Figures**

**Table S1.** Primer sequences for real-time quantitative PCR

| **Source** | **Gene** | **Sequence 5’-3’ ( forward)** | **Sequence 5’-3’ ( reverse)** |
| --- | --- | --- | --- |
| Mouse | MCP-1 | TCACCTGCTGCTACTCATTCACCA | TACAGCTTCTTTGGGACACCTGCT |
|  | ICAM-1 | GCCTTGGTAGAGGTGACTGAG | GACCGGAGCTGAAAAGTTGTA |
|  | VCAM-1 | TGCCGAGCTAAATTACACATTG | CCTTGTGGAGGGATGTACAGA |
|  | β-actin | TGGAATCCTGTGGCATCCATGAAAC | TAAAACGCAGCTCAGTAACAGTCCG |
| Rat | MCP-1 | GTCACCAAGCTCAAGAGAGAGA | GAGTGGATGCATTAGCTTCAGA |
|  | ICAM-1 | AGATCATACGGGTTTGGGCTTC | TATGACTCGTGAAAGAAATCAGCTC |
|  | VCAM-1 | TTTGCAAGAAAAGCCAACATGAAAG | TCTCCAACAGTTCAGACGTTAGC |
|  | β-actin | AAGTCCCTCACCCTCCCAAAAG | AAGCAATGCTGTCACCTTCCC |

**Figure S1.** C66 and SP600125 treatment attenuated the over-expression of adhesion molecules in diabetic mouse kidneys. A-C. The total RNA in kidney tissues of six groups in the supplemental *in vivo* study was extracted respectively, and was performed for RT-qPCR analysis to detect the mRNA level of VCAM-1 (A), ICAM-1 (B), and MCP-1 (C). The mRNA levels (mean ± SD) are expressed as a ratio of β-actin. n=5-8/group). D-F. The total protein in kidney tissues of six groups in the supplemental in vivo study was extracted respectively, and was performed for Western blotting analysis to detect the protein level of VCAM-1 (D), ICAM-1 (E), and MCP-1 (F). The column figures show the normalized optical density from the blot data (4-6 mice in each group, * *p*<0.05, ***p*<0.01, v.s. DM group).

**Figure S2.** C66 and SP600126 treatment decreased renal injuries in diabetic mice in the supplemental *in vivo* study. A. Plasma creatinine levels were determined and normalized by that of one mouse in Con group (*p<0.05). B. H&E staining was used for analysis of histological abnormalities. Six representative figures of histological abnormalities are shown. C. Masson staining was used for analysis of glomerular fibrosis. Six representative figures are shown (×400).


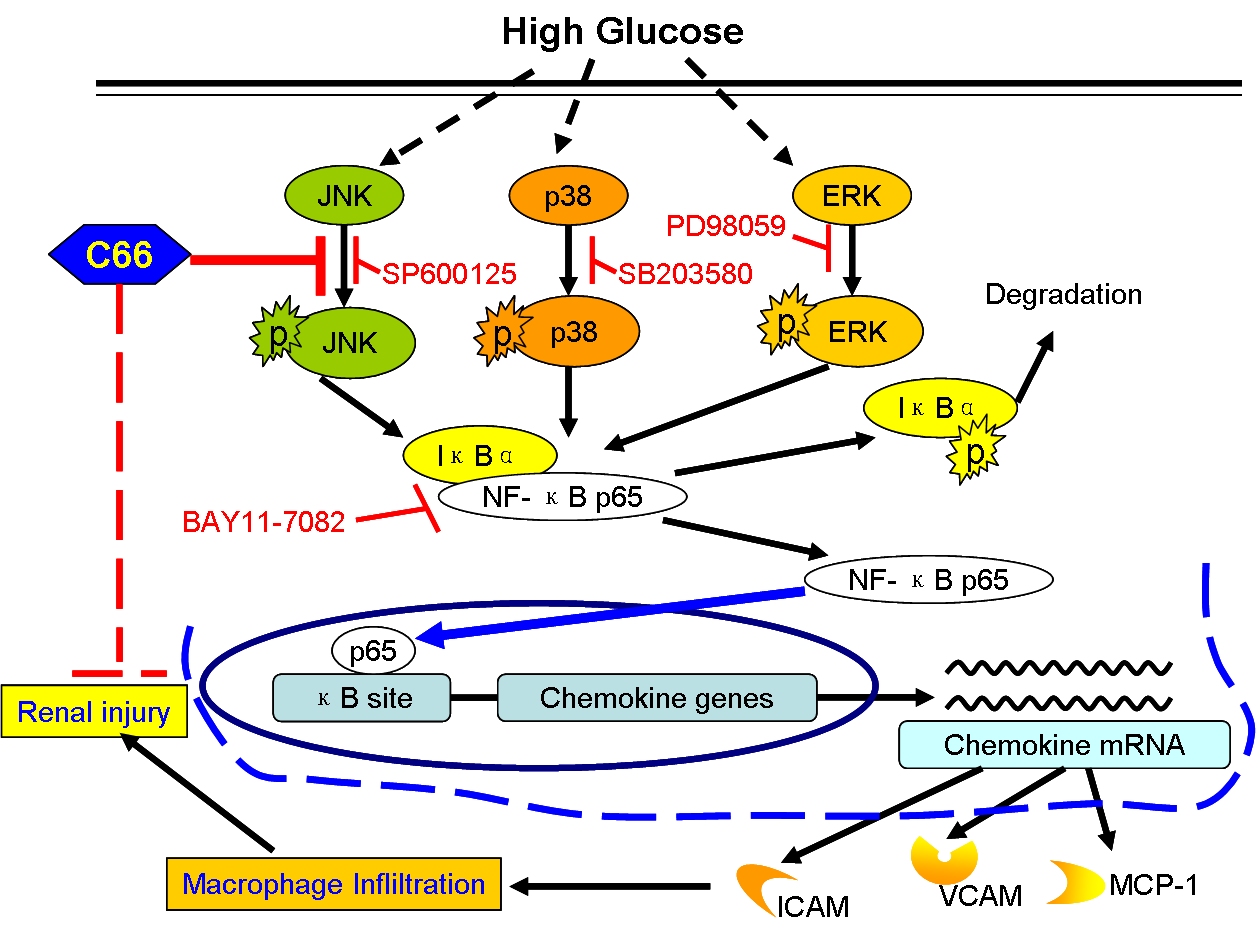


**Figure S3**. A schematic illustration for the prevention of C66 from HG-induced macrophage infiltration and renal injury.
